# Resilience offers escape from trapped thinking on poverty alleviation

**DOI:** 10.1126/sciadv.1603043

**Published:** 2017-05-03

**Authors:** Steven J. Lade, L. Jamila Haider, Gustav Engström, Maja Schlüter

**Affiliations:** 1 Stockholm Resilience Centre, Stockholm University, 106 91 Stockholm, Sweden.; 2 Fenner School of Environment and Society, The Australian National University, Canberra, Australian Capital Territory 2601, Australia.; 3 The Beijer Institute of Ecological Economics and Global Economic Dynamics and the Biosphere, The Royal Swedish Academy of Sciences, 104 05 Stockholm, Sweden.

**Keywords:** Poverty trap, dynamical systems, Development, social-ecological system, agricultural system, environmental degradation, culture

## Abstract

The poverty trap concept strongly influences current research and policy on poverty alleviation. Financial or technological inputs intended to “push” the rural poor out of a poverty trap have had many successes but have also failed unexpectedly with serious ecological and social consequences that can reinforce poverty. Resilience thinking can help to (i) understand how these failures emerge from the complex relationships between humans and the ecosystems on which they depend and (ii) navigate diverse poverty alleviation strategies, such as transformative change, that may instead be required. First, we review commonly observed or assumed social-ecological relationships in rural development contexts, focusing on economic, biophysical, and cultural aspects of poverty. Second, we develop a classification of poverty alleviation strategies using insights from resilience research on social-ecological change. Last, we use these advances to develop stylized, multidimensional poverty trap models. The models show that (i) interventions that ignore nature and culture can reinforce poverty (particularly in agrobiodiverse landscapes), (ii) transformative change can instead open new pathways for poverty alleviation, and (iii) asset inputs may be effective in other contexts (for example, where resource degradation and poverty are tightly interlinked). Our model-based approach and insights offer a systematic way to review the consequences of the causal mechanisms that characterize poverty traps in different agricultural contexts and identify appropriate strategies for rural development challenges.

## Teaser

The complex roles of nature and culture in poverty traps call for diverse and often transformative poverty alleviation strategies.

## INTRODUCTION

Governments worldwide recently agreed to “end poverty in all its forms everywhere” by 2030 with minimal impact on Earth’s life-support systems (*1*). The interplay between development and the environment is particularly relevant for rural agricultural landscapes, which account for 78% of the global poor (*2*). As the world embarks on this momentous challenge, there is an urgent need to take stock of the paradigms that both support and constrain contemporary research and policy on poverty alleviation (*3*). The concept of the poverty trap, in which self-reinforcing mechanisms maintain poverty (see glossary in Box 1), has gained widespread popularity in both academic literature (*4*–*6*) and policy-making (*7*, *8*) on rural poverty. Although the poverty trap concept has directed welcome attention to the fact that poverty can persist, limitations to the poverty trap concept constrain research and policy on integrated poverty alleviation. Here, we argue that the interdisciplinary and integrated lens offered by decades of research on the resilience of social-ecological systems can stimulate significant advancements in research and policy on poverty traps toward more effective responses to rural development challenges.

Box 1 **Glossary of terms.**

Poverty trap: A situation in which self-reinforcing mechanisms “trap” people in poverty (*6*, *71*, *72*).

Attractor: A state (or set of states) of a system toward which nearby states tend over time. In this article, a system state consists of the levels of capitals.

Basin of attraction: The set of states of a system, which tend over time toward the same attractor. We use basins of attraction to visually represent the dynamics of models with two or more capitals.

Physical capital: A manufactured asset that can increase economic output (*58*).

Natural capital: The natural resource stocks from which resource flows and services are derived for human well-being (*73*).

Cultural capital: Values and knowledge transmitted between people and through generations (*74*). Here, we refer specifically to values and knowledge related to use of natural resources (*32*).

Social-ecological system: Integrated system of ecosystems and human society with reciprocal feedback and interdependence. The concept emphasizes that human society is embedded in ecosystems (*20*, *32*).

Context: For the purposes of the models in this paper, the set of social-ecological relationships (as defined in Table 1) that hold in a particular case and thereby determine how capitals interact and vary over time. Here, we are particularly concerned with social-ecological relationships related to how agricultural practices affect the environment (for example, poor people do or not degrade the environment).

Resilience thinking: A loosely organized cluster of concepts and tools for understanding and managing change in complex social-ecological systems (*20*, *21*, *28*). Resilience is generally taken to comprise three capacities: persistence, adaptability, and transformability (*20*).

Limitations of the poverty trap concept affect both understanding of persistent poverty and strategies designed to alleviate poverty. First, although the multidimensional nature of poverty is well known (*9*, *10*), poverty traps are usually characterized in terms of a single dimension such as assets (*11*, *12*). Although social mechanisms, for example, are sometimes incorporated into poverty trap models, the complex dynamics that can occur in multidimensional poverty traps need to be better investigated (*13*–*15*). Second, although the poverty traps concept is not always explicitly invoked, the idea that a sufficiently large injection of assets or other aid can move rural people out of poverty—by “pushing” them over the barrier between poverty and economic growth—is pervasive. Yet, excessive focus on asset inputs, such as finance or technology, risks ignoring well-established knowledge about more endogenous and inclusive poverty alleviation pathways (*16*) and can lead to increased resource degradation or the loss of biological and cultural diversity (*17*). Development interventions that overlook such endogenous and contextual factors may therefore exacerbate rather than alleviate poverty (*18*, *19*).

Moving beyond a single-dimensional understanding of rural poverty traps is urgently required (*13*–*15*) but severely challenging. The mechanisms that create and maintain persistent poverty interact in complex ways, depend strongly on context, are often highly contested, and may require qualitatively different alleviation pathways. Resilience research has developed concepts and insights, collectively known as “resilience thinking,” to deal with similar challenges in natural resource management (*20*, *21*). Here, we apply resilience thinking to poverty traps as follows. (i) To address the complex, multidimensional social-ecological relationships that give rise to persistent poverty in poor agricultural communities, we review relationships between poverty and the environment that are commonly observed or assumed in rural agricultural landscapes. The set of social-ecological relationships that apply in a particular case constitutes the “context” for that case (Box 1). (ii) The diverse dynamics that arise from the different relationships between agricultural production, environmental degradation, and cultural aspects of rural communities call for diverse alleviation strategies. To understand and compare how different poverty alleviation pathways can affect poverty traps, we develop a classification of alleviation pathways based on concepts of social-ecological change from resilience thinking. (iii) We use these advances to design a series of stylized, multidimensional poverty trap models. From these models, we obtain a series of novel insights that provide clear conditions on how different outcomes of different poverty alleviation pathways emerge from different rural agricultural landscapes. Through these three steps, we offer a systematic way to investigate the consequences of the causal mechanisms that characterize poverty traps in different contexts and identify appropriate strategies for rural development challenges.

## Poverty alleviation through a resilience lens

Resilience has become a popular concept in development policy (*22*, *23*), where it is commonly viewed as a property of individuals or communities that can be “built” through investments and used to measure the effectiveness of these interventions. The view that resilience is an outcome or a property that can be measured, although useful for some purposes (*24*), risks dangerously simplifying and neglecting aspects of the complex social-ecological dynamics that characterize most development situations (*25*). The conceptual insights developed by resilience thinking, in contrast, are well suited to understanding how poverty traps occur and are maintained, given the common conceptual basis of attractors, thresholds, and other nonlinear dynamics shared by poverty trap models and resilience thinking (*13*). We apply insights on social-ecological interactions, regime shifts, and transformations from resilience thinking to the study of poverty traps and alleviation pathways. Our work advances previous applications of resilience thinking to poverty traps by going beyond the assumption that poverty degrades the environment (*26*), heuristics that predetermine the properties of the poverty trap (*27*–*29*), and sketches of hypothetical multidimensional poverty trap models (*13*, *15*). We use resilience thinking to build multidimensional, process-based dynamical models of poverty traps in rural agricultural communities. The models allow properties of poverty traps and outcomes of poverty alleviation to emerge from multiple poverty-environment relationships and multiple poverty alleviation pathways

### Social-ecological relationships in rural poverty

**Fig. 1:**
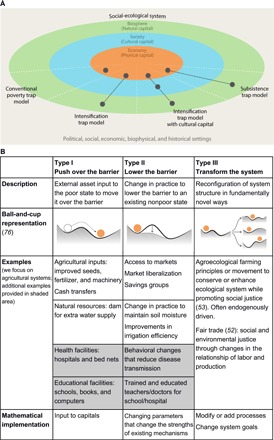
A resilience approach to poverty traps. (**A**) Dealing with the interactions between multiple dimensions of poverty is critical to understanding and alleviating poverty. Here, we focus on humans and nature as part of interlinked social-ecological systems, in which economic activity is dependent on society and the biosphere. To assess how these interlinkages affect alleviation of rural poverty, we construct a series of multidimensional poverty trap models (gray dots and lines). In these models, we operationalize the dimensions of “biosphere,” “society,” and “economy” using capitals. (**B**) Classification of poverty alleviation pathways based on resilience thinking. This classification is used to implement different poverty alleviation pathways in our multidimensional poverty trap models. The three types are neither exhaustive nor mutually exclusive; any specific alleviation strategy is likely to combine features of multiple types. [Image credit: J. Lokrantz/Azote]

Human-dominated agricultural landscapes are not only home to the majority of the world’s poor (*2*) but are often refugia for biological and cultural diversity (*30*). In these landscapes, ecosystems, people, and their traditions, languages, and practices are tightly interlinked (*31*) as so-called social-ecological systems (Box 1) (*32*). Resilience research demonstrates that these relationships are crucial in shaping the dynamics of social-ecological systems (*33*), and emphasizes that the biosphere is the foundation for all social and economic activity (Fig. 1A) (*20*). To build multidimensional poverty trap models that assess how social-ecological relationships and perceptions about them influence poverty alleviation pathways, we first review assumptions and observations about the relationships between poverty and the environment in rural poor landscapes. These relationships are complex, dynamic, and disputed (*34*).

That poverty can lead to an increased rate of environmental degradation (Table 1) is an assumption that has dominated discourse on the poverty-environment nexus (*35*, *36*) and was clearly articulated in the Brundtland Commission (*37*). For example, declining yields in severely degraded landscapes, such as sub-Saharan Africa (*26*), force the poor to farm increasingly small plots of land ever more intensively, reinforcing continued degradation of the land. In some frontier regions, poor people may increasingly encroach on primary forests to clear more land for agriculture (*38*). Poverty alleviation strategies that are based on the assumption that poverty degrades the environment often focus on intensifying household agricultural production to alleviate poverty and reduce environmental degradation (*39*).

**Table 1:** Commonly assumed or observed social-ecological relationships in local, agricultural, developing world contexts. We emphasize that none of these assumptions are “right” or “wrong” but will apply in some cases and not in others, or offer a different level of explanation for the same relationship. This literature review is intended to be representative rather than exhaustive. We use some of these assumptions to build multidimensional poverty trap models.

**Relationship**	**Assumption/observation**	**Use in models**
Poverty and environmental degradation	Poor people degrade the environment: Poor people are heavily resource- dependent; they have no other option than to exploit and degrade natural resources (*37*, *77*). This relationship is empirically observed (*26*, *78*).	Subsistence trap model (Fig. 3, C to E)
	Poor people do not degrade the environment: No evidence for causal relationship between poverty and biodiversity loss (*79*, *80*). Poor people are often those with strong traditions of agricultural practice, which creates and maintains biodiversity and other features of agricultural landscapes (*31*, *32*, *81*). Medium or large landholders can be the primary perpetrators of deforestation (*82*). See also justification for intensification degrades.	Intensification trap model [Figs. 2 (D to I) and 3 (A and B)]
	Poor people degrade the environment but this is due to political and socioeconomic relations: Environmental degradation by the poor is caused by consumption patterns of the rich. Poverty itself is politically and historically caused (for example, colonialism) (*35*, *77*).	—
Conventional agricultural intensification and environmental degradation	Intensification degrades: Conventional agricultural intensification degrades the environment (*83*, *84*).	Intensification trap model [Figs. 2 (D to I) and 3 (A and B)]
	Environmental effects of intensification are not considered: Short-term productivity gains outweigh possible long-term effects on the environment (*85*, *86*).	By omission of natural capital, conventional poverty trap model (Fig. 2, A to C)
Sustainable intensification and environmental degradation	Sustainable intensification works: Sustainable intensification can produce more output from the same area of land while reducing negative environmental impacts (*66*, *87*, *88*).	Subsistence trap model (Fig. 3, C to E)
	Sustainable intensification can have unintended consequences: “In practice [sustainable intensification] can mean business-as-usual intensive farming with slight modifications to try and tackle the growing environmental crises caused by industrial agriculture (*89*).”	—
Economic development and environmental degradation	Environmental Kuznets curve holds: Industrialization initially increases environmental degradation, until some point where technology improves and degradation decreases (*90*, *91*).	—
	Environmental Kuznets curve does not hold: The curve is generally not supported by empirical evidence; it assumes an industrial development trajectory; it does not consider effects on finite global resources (*92*, *93*).	—
Traditional knowledge and environmental conservation	Traditional knowledge and practice conserve the environment: Traditional knowledge and practices have coevolved with the environment in some landscapes. Local or indigenous peoples often have a relationship with natural resources that enable sustainable management (*17*, *32*, *41*, *94*).	Intensification trap model with cultural capital [Figs. 2 (G to I) and 3 (A and B)]
	People should decouple from agricultural land to conserve the environment: Intensive development and technology are necessary to achieve sustainable development. Humans should be decoupled from the land through rapid urbanization (*95*).	—

In contrast, the conventional high-input agricultural systems often recommended to increase production can also degrade the biophysical systems on which agricultural production depends (Table 1). Moreover, in the agrobiodiverse landscapes that often characterize poor agricultural contexts, poor people often do not degrade the environment but rather actively create and maintain the diverse agricultural landscape on which they have long depended (Table 1). Examples of such landscapes include Transylvania, which is the poorest region of Eastern Europe (*40*), and some tropical agricultural landscapes (*41*). In these situations, poverty alleviation pathways involving agricultural intensification may lead to an increased, rather than decreased, rate of degradation of natural resources.

In rural landscapes, social-ecological relationships are mediated by land management knowledge and practices (Table 1) (*32*). For example, in central Asia’s Pamir Mountains, seed varieties that are pest- and drought-resistant, grow in poor soils, replenish soil nutrients, and prevent soil erosion have coevolved with traditional cultivation practices, culture, and language (*42*). Development interventions that increase agricultural productivity and marketability through introduced seed varieties often risk the loss of local seeds and the corresponding culture and language that maintain a sustainable agricultural landscape. In another example, the practice of growing subsistence crops alongside coffee in plantations in Costa Rica ensures the survival of pollinators necessary for producing coffee beans (*43*). When farmers switch to high-input monocultures, opportunities to engage in these practices and the knowledge associated with them can be lost (*44*).

### Conceptualizing poverty alleviation pathways using resilience thinking

The poverty trap concept was developed to explain the existence of multiple stable states of economic development, particularly the existence of persistent poverty (*45*, *46*). Responses to poverty traps have therefore focused on lifting poor people over asset thresholds between poor and nonpoor states. From origins in the nonlinear dynamics of ecosystems (*47*), resilience thinking similarly seeks to explain multiple attractors (Box 1) in social-ecological systems. We use the multiple concepts of social-ecological change that resilience thinking has developed—specifically, regime shifts and transformations—to understand three different ways in which poverty alleviation pathways can affect poverty traps (Fig. 1B).

Regime shifts are large, abrupt, and persistent shifts across thresholds into different attractors in social-ecological systems (*48*, *49*). Resilience thinking identifies two broad types of regime shifts on which we base our first two types of poverty alleviation pathways: (i) A sudden external influence pushes the system “over the barrier” (or threshold) between two attractors. Such an external influence could be asset inputs such as money, farming equipment, or novel seeds. We label such a strategy that aims to push people over the barrier between poor and nonpoor attractors as a type I alleviation pathway. In a conventional development economics model called the savings trap (*45*), the barrier is an asset threshold below which people are unable to save a sufficient proportion of production to invest in improving production (Box 2). (ii) Changes in human practices or biophysical processes within the social-ecological system lead to “lowering the barrier” to an already existing developmental trajectory. We label such changes as a type II alleviation pathway. For a poor agricultural community, a type II intervention could include improved market access that enables an increase in savings from agricultural production, a women’s saving group, a community-based natural resource management group, or high-quality public education programs.

Box 2 **Conventional multiple-equilibrium poverty trap model.**

“S curve” models resulting from Solow growth models are the basis for the poverty trap models popularly in use today (*72*) at household (*95*, *96*) and at national scales. In these models, nonlinear asset dynamics give rise to multiple asset equilibria.

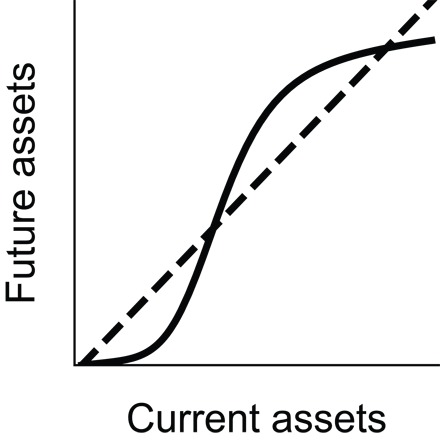

**Fig. 2:**
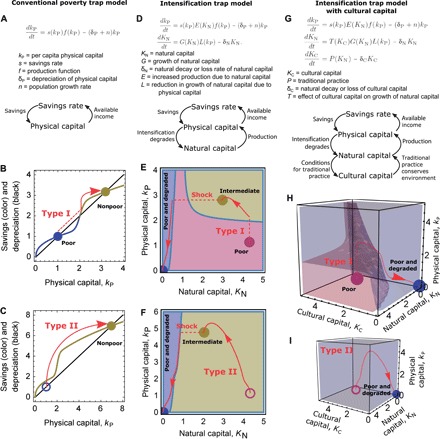
Conventional alleviation pathways lead to deleterious consequences in intensification trap models. Equations and loop diagrams representing the feedbacks that reinforce poverty in the conventional poverty trap model (**A**) and two- and and three-dimensional intensification trap models (**D** and **G**). These models are subject to type I (**B**, **E**, and **H**) and II (**C**, **F**, and **I**) interventions. For the conventional poverty trap model, we plot the functional forms for the savings and depreciation terms; fixed points are the intersections of these curves. For the higher-dimensional models, we plot attractors (colored discs) and their basins of attraction (colored areas or volumes). Trajectories associated with different alleviation pathways (red arrows) and attractors before type II interventions (unfilled colored circles) are shown. Full specification of the models’ qualitative assumptions is provided in table S1, and their mathematical form is provided in Supplementary Methods.

As a starting point, we use a model of the savings trap (*45*) applied to a household scale. In a savings trap, people are unable to save a sufficient fraction of income to invest in improved methods that would increase production. In this model (Fig. 2A), a sufficiently strong “push over the barrier” (type I) pathway involving farm inputs, such as fertilizers and improved seeds (Fig. 2B), will allow escape from the poverty trap. A “lower the barrier” (type II) pathway involving institutional change such as market reform can both remove the poor attractor altogether and increase the level of the remaining nonpoor attractor (Fig. 2C). The savings trap is a standard model of a multiple equilibrium poverty trap, as opposed to a single equilibrium model (see main text).

Our third type of alleviation pathway builds on the concept of transformation in social-ecological systems. In resilience thinking, transformation is a structural change that “recombines existing elements of a system in fundamentally novel ways” (*50*). Effective transformations often (i) are intentional, as opposed to change triggered by external shocks; (ii) may be supported by an external type I or II intervention but are led by actors endogenous to the system being transformed; (iii) involve these actors setting priorities justified by narratives fundamentally different to the status quo (*51*); and (iv) lead to change across multiple levels of society (*20*, *50*). We refer to alleviation pathways with some or all of these characteristics as a type III or “transform the system” alleviation pathway. For example, agroecological farming that improves production by combining modern farming techniques with traditional cultivation practices, when combined with farmer choice over whether and how to interact with global food markets, could lead to transformations that achieve more social justice and reduced poverty (*52*, *53*).

## Effective pathways for escaping multidimensional poverty traps

Moving beyond the one-dimensional poverty trap concept and asset input responses requires poverty trap models that can incorporate multiple social-ecological relationships and can assess diverse poverty alleviation pathways. Here, we use our review of relationships (Table 1) and classification of pathways (Fig. 1B), which were structured by resilience thinking, to construct stylized dynamical system models of multidimensional poverty traps. In the models, the effectiveness of different alleviation pathways (judged by whether the social-ecological system reaches and persists in a nonpoor state) emerges from the social-ecological relationships present in that situation. The relationships between poverty, environment, and culture (Table 1), whose effects we investigate, are supplemented with standard assumptions from economic and ecological modeling about poverty traps and ecosystem dynamics (table S1). These stylized dynamical models are useful as a reasoning tool to understand how the effectiveness of different alleviation pathways may be causally affected by different factors, as opposed to the more common use of dynamical models for quantitative prediction and hypothesis testing.

To build multidimensional poverty trap models, we begin from a conventional multiple-equilibrium poverty trap model (Box 2) and progressively add biophysical and cultural features of social-ecological systems in poor agricultural landscapes (see Materials and Methods). Conventional poverty trap models usually use aggregated variables such as accumulated assets, wealth, or well-being; we take a similar aggregated, stylized approach and represent the economic, biophysical, and human dimensions in terms of capitals (Fig. 1A) (*54*). We initially make the assumption that the main goal of poverty alleviation strategies is to increase physical capital to demonstrate how complex social-ecological relationships can complicate even this narrow view of development. Although social, political, economic, and biophysical interactions at a variety of scales influence the formation and persistence of poverty traps (*55*), we restrict this study to the household or farm scale because this is the scale at which social-ecological interactions between farming practices and ecosystem dynamics occur.

Although the multiple equilibrium poverty trap (Box 2) is the most commonly used model, and we therefore use it as the starting point for our models, there is debate over whether multiple equilibrium poverty traps actually exist (*12*, *16*). In situations where a single equilibrium poverty trap model is more appropriate (*56*), a type I push over the barrier intervention cannot work because there is no alternative attractor to push into, but our results regarding type II and III strategies do not qualitatively change.

### Interventions that ignore nature and culture can reinforce poverty

In a conventional single-dimensional, multiple-equilibrium poverty trap (Box 2 and Fig. 2A), asset inputs such as cash, technology, artificial fertilizer, or pesticides (type I alleviation pathway) succeed once the input is sufficiently strong (Fig. 2B). Consider a situation (Fig. 2D and table S1B) where “poor do not degrade” but (agricultural) “intensification degrades” (Table 1) the natural capital that supports agricultural production, such as soil health, on- and off-farm biodiversity, and availability of landrace seeds (*57*, *58*). In this model, a sufficiently strong push over the barrier (type I) intervention on physical capital (for example, using farm inputs) can reach an attractor with increased physical capital. Although increased physical capital increases production, degradation of natural capital associated with higher physical capital in this model makes this state highly vulnerable to stochastic shocks such as natural disasters or price fluctuations. Responses to a large shock could include returning to the initial attractor or, if the natural capital is subject to tipping points, degenerating into an attractor of extremely low physical and natural capital (Fig. 2E). Because intensification led to alleviation failure or even negative consequences, we refer to this model as an intensification trap, similar to the pesticide trap (*59*) or modernization trap (*44*).

Explicitly recognizing the role of culture leads to an even more pessimistic view about the possible effectiveness of type I pathways, such as asset inputs. Where traditional agricultural knowledge and practice, which we implement as cultural capital (Box 1), supports the maintenance of natural capital (Fig. 2G and table S1C), any type I pathway that succeeds in overcoming the physical capital threshold (Fig. 2H) results in very low levels of all three capitals, without even any intermediate attractor. A vicious cycle occurs in which decline of traditional practice reduces the level of natural capital, which, in turn, leads to further decline of traditional practice. Three mechanisms in the model now interlock to obstruct poverty alleviation (Fig. 2G): (i) the physical capital threshold from the original savings trap, (ii) the degradation of natural capital due to intensification, and (iii) the decline of traditional practice now described.

These dynamics in our intensification trap model reflect frequently observed development failures (*16*, *18*, *60*). The failures of asset inputs are sometimes used to promote alternative pathways such as market liberalization (*16*). Improved market access, implemented as a lower the barrier (type II) pathway, succeeds in a conventional poverty trap model (Fig. 2C) but, like type I, also fails in the intensification trap models (Fig. 2, F and I). As in type I, intensification leads to decreases in natural capital, which renders the natural capital highly vulnerable to shocks.

We conclude that both external aid (type I) and market liberalization strategies (type II) may lead to unexpected negative consequences in situations where agricultural intensification results in degradation of natural capital, such as in some agrobiodiverse landscapes. That the situation becomes even worse once the role of cultural capital is recognized reflects the practical difficulty of alleviating poverty that persists because of multiple interlocking mechanisms (*61*).

### Effective poverty alleviation can require transformations that open new pathways

Our previous models (Fig. 2), in which type I and II pathways failed, indicate that transformations (type III pathways) that enable development while avoiding environmental and cultural degradation may be critical to alleviate poverty. We now model the local-scale effects of a transformative pathway in which farmers choose to combine conventional agricultural production with traditional agricultural practices (*62*).

**Fig. 3:**
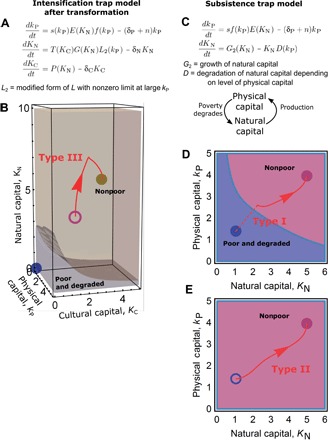
Effective poverty alleviation is context-dependent. Intensification trap model from Fig. 2G after a transformation (**A**) as part of a transform the system (type III) pathway (**B**). Function definitions from Fig. 2 also apply. Subsistence trap model (**C**) after type I (**D**) and II (**E**) interventions. Model dynamics are shown using basins of attraction and alleviation trajectories, as in Fig. 2. Full specification of the models’ qualitative assumptions is provided in table S1, and their mathematical form is provided in Supplementary Methods.

Our model assumes that farmers devote at least a portion of their otherwise intensively farmed land to local crops maintained through traditional practice (Fig. 3A and table S1D) (*17*). This transformation pathway, which could be supplemented by physical capital inputs such as selective phosphorus application, replaces the previous poor attractor (Fig. 2H) with a new attractor (Fig. 3B). In the new attractor, production is increased because of a large increase in natural capital. We label this new attractor as “nonpoor” because long-term agricultural production increased. Rather than increasing production through inputs of physical capital, the transformation delivers increased production primarily due to increases in natural capital, supported by improvements in cultural capital. Vulnerability to shocks also decreases with a much larger basin of attraction.

A transformative pathway involving a careful integration of modern approaches with traditional practice may therefore be successful in the context of an intensification trap, as, for example, some food sovereignty approaches have demonstrated (*17*, *62*, *63*). In practice, several factors may complicate the feasibility of agricultural transformations when multiple interacting scales are considered (*64*). Nevertheless, we conclude that the scope of alleviation strategies treated by poverty trap models can and must be expanded to include strategies outside the mainstream, for example, transformations based on agroecological practices (*65*).

### Asset inputs are effective in some contexts

We have shown how, in contexts where an intensification trap exists, a transformation may be necessary to escape poverty. We do not wish to suggest, however, that type III pathways are the only useful approach to poverty alleviation. In this section, we briefly explore how more conventional type I and II strategies may be effective.

Consider a situation (Fig. 3C and table S1E) where a “poor people degrade the environment” (Table 1) relationship holds and agricultural intensification does not lead to increased environmental degradation (“sustainable intensification works”; Table 1). A “subsistence trap” can emerge in which low levels of natural and physical capital reinforce each other (Fig. 3D). A push over the barrier (type I) strategy involving agricultural asset inputs, such as fertilizer inputs in areas with nutrient-poor soils (*66*), may lead to escape from a subsistence trap (Fig. 3D). In the model, increased production triggered by agricultural inputs leads to decreased exploitation of natural capital, breaking the feedback loop of the subsistence trap (Fig. 3C).

A sufficiently strong lower the barrier (type II) pathway that improved the renewal rate of natural capital through a change in practice may also work (Fig. 3E). These predicted successes are confirmed by empirical findings. For example, in field experiments in Tanzania (*26*), conservation tillage in combination with supplemental irrigation improved replenishment of soil moisture, broke a pattern of degradation of on- and off-farm resources, and yielded large increases in crop yield. We conclude that asset inputs that are suitably matched to specific social and agroecological conditions may be appropriate poverty alleviation pathways, especially in highly degraded or frontier landscapes, where poverty can be associated with increased environmental degradation.

Our model results, that type I or II alleviation pathways may be effective for escaping a subsistence trap, only apply, however, to interventions that are consistent with the assumptions of the subsistence trap model (“sustainable intensification works” and “poor people degrade” in Table 1). For example, choices of physical capital inputs that are not well adapted to local ecological, cultural, and socioeconomic conditions could lead to results like those seen in the intensification trap models. Furthermore, we do not consider here the role of cultural capital in a subsistence trap, which we showed in the intensification trap can be an important factor in whether a poverty trap is escaped or maintained.

## Implications for policy and research

**Fig. 4:**
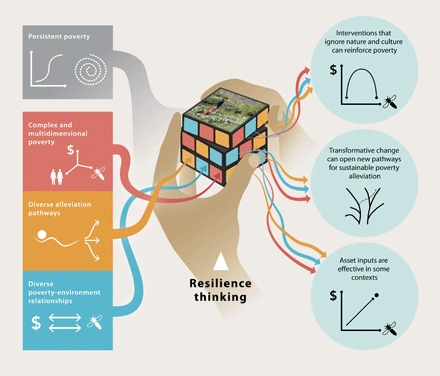
Solving the poverty-environment puzzle. Using resilience thinking, we integrate complex interactions among multiple dimensions of poverty, diverse poverty alleviation pathways, and diverse poverty-environment relationships into the poverty trap concept. From multidimensional poverty trap models based on these inputs, insights on effective poverty alleviation pathways emerge. [Image credit: E. Wikander and E. Wisniewska/Azote]

Global sustainability challenges, such as those outlined by the United Nations’ 2030 Agenda for Sustainable Development, are complex and interconnected (*3*). For example, the Sustainable Development Goals (SDGs) call for alleviation of all poverty everywhere within the next 15 years while not endangering the biophysical systems on which humanity’s continued prosperity depends. Yet, conceptual frameworks and measurement tools remain unable to satisfactorily deal with this interconnected complexity. Stylized models based on resilience thinking provide a novel way to approach this “poverty puzzle” by integrating thinking on complex and multidimensional poverty, diverse alleviation pathways, and diverse poverty-environment relationships (Fig. 4).

Our analysis of the interdependencies between physical, natural, and cultural capitals in shaping poverty traps and potential poverty alleviation pathways provides three key insights for policy-makers (Fig. 4, right column). First, it is impossible to understand persistent poverty without explicitly accounting for the interactions between the multiple dimensions of social-ecological systems. Ignoring relationships between agricultural production, nature, and culture can reinforce poverty, such as in often-marginalized agrobiodiverse contexts where biodiversity sustains livelihoods and healthy agroecosystems. Improvements to agricultural productivity intended to achieve the first and second SDGs (“no poverty” and “end hunger”) could therefore, in these contexts, risk reinforcing rather than alleviating poverty. Second, it is vital to consider a more nuanced range of alleviation pathways, beyond conventional asset inputs, to overcome poverty traps. Transformations that open new developmental pathways can be critical for effective poverty alleviation (*67*). Here, we studied the local consequences of a transformative pathway based on combining conventional agricultural production with traditional practice. Last, conventional development interventions may be useful in some contexts [such as highly degraded frontier landscapes (*38*)] and should not be ignored. Researchers and development practitioners alike could use stylized models, such as those we developed here, to interrogate shared or conflicting assumptions about mechanisms that maintain poverty and affect development strategies in different contexts.

Moving development practice and thinking beyond a one-dimensional understanding of persistent poverty will also require new theoretical advances regarding the causal mechanisms that underpin both poverty traps and potential alleviation strategies. We call for a new research agenda that not only focuses on the multidimensional nature of poverty itself but also strengthens efforts to understand the interplay of multiple physical, natural, and cultural factors that shape the relative success or failure of development interventions in agricultural systems across the developing world. First, there is a need to systematically and critically reflect on how the relationships that create and maintain poverty differ according to context. Here, we focused on relationships among economic, biophysical, and cultural dimensions of poor agricultural communities, in particular on the implications of agricultural intensification in agrobiodiverse contexts. Other critical factors that should be addressed in future research include (i) interactions between other dimensions such as health and education (*14*), (ii) historical legacies that maintain poverty traps and may have been instrumental in establishing them (*17*, *68*–*70*), and (iii) cross-scale interactions involving power and inequality and governance at multiple levels (*17*, *19*, *34*). Second, new research is needed to systematically study how context, as represented by social-ecological relationships that characterize a specific situation, affects poverty alleviation. Building on existing poverty trap models, our stylized models make a step in this direction by exposing the link between qualitative assumptions about social-ecological relationships and qualitative observations regarding the effectiveness of alleviation strategies.

The poverty trap has proven a useful concept in popularizing efforts to combat persistent poverty across many parts of the developing world. However, one-dimensional conceptualizations of poverty traps encourage a narrow range of alleviation responses. Resilience thinking can progress research and policy on poverty alleviation by helping to understand the complex dynamics of the social-ecological environments in which the rural poor live. Combining resilience thinking concepts with simple models as proposed here allows for systematic navigation among the different alleviation responses, from asset inputs to transformative change, that are required for integrated sustainable development.

## MATERIALS AND METHODS

Here, we summarize the mathematical formulation of the poverty trap models used in Figs. 2 and 3. Full specification of the models’ qualitative assumptions is provided in table S1; full mathematical form and reasoning behind the translation of the qualitative assumptions into mathematical form are provided in Supplementary Methods. Basins of attraction in the figures were calculated in Mathematica by clustering final states of grids of initial conditions.

### Conventional poverty trap model

We followed the conventional implementation of a savings trap using a Solow growth model (*71*), in which accumulation of physical capital by saving a proportion of production counteracts decreases of per capita physical capital due to depreciation and population growth (Fig. 2A and table S1A). To produce a multiple-equilibrium poverty trap model, we chose a capital-dependent savings rate in which savings rates increased above some level of physical capital (*56*).

### Intensification trap models

We added a “natural capital” state variable to the conventional poverty trap model (Fig. 2D). In the model, natural capital increases agricultural production by mimicking a two-input Cobb-Douglas production function and endogenously grows using a Holling type III function commonly used for biomass growth, but this growth rate reduces at high levels of physical capital (table S1B). We also added a “cultural capital” state variable (Fig. 2G and see also glossary in Box 1). In this model, traditional agricultural knowledge and practice enhance the growth rate of natural capital and are themselves maintained by the presence of natural capital (table S1C). In a third variant of the intensification trap model, the intensification trap model after transformation (Fig. 3A), we modified the growth rate of natural capital to model the effects of a situation in which farmers can maintain some traditional crops with traditional practices even at high levels of physical capital (table S1D).

### Subsistence trap model

In this model (Fig. 3C), natural capital underwent increased degradation at low levels of physical capital (table S1E), which we modeled using an (inverted) sigmoidal function of physical capital. Natural capital grew logistically and enhanced agricultural production, which we modeled by mimicking a two-input Cobb-Douglas production function. Because feedback between physical and natural capital is sufficient to produce multiple stable states, we removed the savings trap feedbacks associated with variable savings rate.

## Supplementary Material

http://advances.sciencemag.org/cgi/content/full/3/5/e1603043/DC1

## SUPPLEMENTARY MATERIALS

Supplementary material for this article is available at http://advances.sciencemag.org/cgi/content/full/3/5/e1603043/DC1 Supplementary Methods table S1. Qualitative model assumptions. References (*98*–*101*)
