# Supplementary material for: Resilience offers escape from trapped thinking on poverty alleviation
Source: Sci Adv. 2017 May 3;3(5):e1603043. doi: 10.1126/sciadv.1603043 (PMC5415336; doi:10.1126/sciadv.1603043)
Supplement: http://advances.sciencemag.org/cgi/content/full/3/5/e1603043/DC1 [file supp_3_5_e1603043__index.html]

Science Advances | Science Advances

## Supplementary Materials

**This PDF file includes:**

- Supplementary Methods
- table S1. Qualitative model assumptions.
- References (*98–101*)

Download PDF

**Files in this Data Supplement:**

- Adobe PDF - 1603043\_SM.pdf
