# Supplementary material for: Resilience offers escape from trapped thinking on poverty alleviation
Source: Sci Adv. 2017 May 3;3(5):e1603043. doi: 10.1126/sciadv.1603043 (PMC5415336; doi:10.1126/sciadv.1603043)
Supplement: http://advances.sciencemag.org/cgi/content/full/3/5/e1603043/DC1 [file 1603043_SM.pdf]

Science Advances

AAAS

advances.sciencemag.org/cgi/content/full/3/5/e1603043/DC1

Supplementary Materials for

Resilience offers escape from trapped thinking on poverty alleviation

Steven J. Lade, L. Jamila Haider, Gustav Engström, Maja Schlüter

Published 3 May 2017, *Sci. Adv.* **3**, e1603043 (2017)
DOI: 10.1126/sciadv.1603043

This PDF file includes:

- Supplementary Methods
- table S1. Qualitative model assumptions.
- References (*98*–*101*)

Supplementary Methods

Here, we provide the mathematical forms for the multidimensional poverty models used in the main text. This document should be read in conjunction with table S1, which provides detailed justifications for the qualitative forms of the models.

We used deterministic models, rather than stochastic models as recently proposed by Barrett and Constas (*13*), to simplify the models' formulation and the presentation of their results. Our deterministic models could easily however be replaced by stochastic variants involving the conditional expectation functions (CEFs). Interventions involving modifications to the CEF may fall into either the *lower the barrier* (type II) or *transform the barrier* (type III) categories, depending on whether the model of the intervention involves a simple change to parameters of the CEF or an entirely new CEF.

All references refer to the reference list in the main text.

1 Conventional poverty trap models

A wide variety of models featuring different mechanisms that cause poverty to persist have been proposed in literature (*73*). Common to all these models that generate multiple equilibria is a non-convexity in the state dynamics. Here, we use the savings trap (*45*) implemented with the Solow growth model as an example of a conventional poverty trap model. The saving rate is assumed to vary with per capita capital in an S-shaped manner, i.e. it is low for low levels of per capita capital, increases, and levels off at high levels. The original Solow model (*71*) makes use of an aggregate production function characterizing economic growth at the nation level, but with a clear micro-economic foundation at the household level. This feature makes it applicable also at the community scale which we are considering here.

1.1 The savings trap model

We specify a model of accumulation of physical capital, the quantity of machines, equipment and structures of various sorts used in production. In the standard formulation of the Solow model, physical capital depreciates at a constant rate of δ_P. We allow for exogenous population growth at a constant rate of n, but disregard technological growth in order to simplify notation (though exogenous technological growth could easily be added to the model similar to that of the original Solow model).

Per capita physical capital k_P evolves as follows

$$\frac{dk_P}{dt} = s(k_P)f(k_P) - (\delta_P + n)k_P$$

where the saving rate s is dependent upon the level of per capita capital. Production per capita and the saving rate are given by

$$f(k_P) = Ak_P^{\alpha_1}$$
$$s(k_P) = s_1 + \frac{s_2 - s_1}{1 + e^{-s_3(k_P - d)}}$$

f is thus a standard neoclassical production function with technology A. To model a saving rate $s(k_P)$ that steps from initial value s_1 up to s_2 at $k_P \approx d$, we used a sigmoid function. This function is the key nonlinearity creating the savings trap.

We chose parameter values that produced a multiple-equilibrium poverty trap model in which there are both poor and non-poor attractors. Parameter values were $A = 10$, $\alpha_1 = 0.4$, $s_1 = 0.1$, $s_2 = 0.2$, $s_3 = 10$, $d = 2$, $\delta_P = 0.5$, $n = 0.5$.

We model a type II alleviation pathway that increases the productivity of the representative farmer or community through an external institutional change by raising the value of A to 16, a sufficiently high value for the poor attractor to disappear. (Alternatively, we could change parameters of the function $s(k_P)$ to represent increases in the returns to savings.) This increase in productivity could arise due to mechanisms such as economic reform leading to more efficient use of resources and thereby an increase in productivity, or improved access to financial markets leading to increased returns on savings.

See table S1A for further detail on the qualitative assumptions behind this model.

2 Intensification trap model

2.1 Physical and natural capital

We now couple natural capital K_N into the previous section's poverty trap equation. We use the equations:

$$\frac{dk_P}{dt} = s(k_P)E(K_N)f(k_P) - (\delta_P + n)k_P$$
$$\frac{dK_N}{dt} = G(K_N)L(k_P) - \delta_N K_N$$

In this equation, natural capital grows at a density-dependent rate $G(k_N)$ that is modified by $L(K_P)$, and lost at proportional rate δ_N. The functions $s(k_P)$ and $f(k_P)$

are as defined above and the other functions were assigned forms

$$E(K_N) = qK_N^{\alpha_2}$$
$$G(K_N) = \frac{bK_N^2}{H^2 + K_N^2}$$
$$L(k_P) = \frac{1}{1 + c_1 k_P^{c_2}}$$

The function $E(K_N)$, representing the contributions of natural capital to production, takes a simple power-law in order to mimic a two-input Cobb-Douglas production function. The natural capital growth function $G(K_N)$ is a conventional Holling type III function commonly used for biomass growth. This functional form is well known to produce dynamics involving tipping points, or regime shifts, in which a sudden collapse occurs that is difficult to reverse. Such dynamics are widely known to occur in natural resources. It is plausible, particularly in the case of agriculture, that a sufficiently severe degradation in the quality of the land will lead to it being unusable.

The growth of natural capital is, in this model, reduced at high levels of physical capital by $L(k_P)$ as per the 'intensification degrades' modelling assumption. We assigned a simple form such that $L(0) = 1$ and $\lim_{k_P \to \infty} = 0$.

Note also that K_N is total natural capital in the social-ecological system of interest, whereas k_P is physical capital is per capita population. The per-capita physical capital k_P is used to indicate the level of industrialisation of farming technologies that are likely to be in use, and thereby the rate of degradation of natural capital. Likewise, we assume that per capita production is affected by per capita physical capital k_P but the state of total natural capital K_N within the system boundaries being considered.

Parameters used were as in previous section with additional parameters $\alpha_2 = 0.4$, $\delta_N = 1$, $b = 10$, $H = 1.5$, $c_1 = 1$, $c_2 = 0.5$, $q = 0.6$ chosen to satisfy the following requirements:

- That there is a non-trivial poor attractor (that is, an attractor that is below the threshold in savings rate $k_P = d$ other than the attractor at zero physical and natural capital)
- That the same change to A as in the conventional poverty trap model (i.e. $A = 10$ to 16), representing a type II intervention, ensures that the poor attractor disappears.
- That the intermediate attractor exists. For some parameter values, no non-poor attractor exists and any type I intervention on physical capital will immediately

return to the poor attractor, which does not affect our main conclusion about the failure of this type of intervention.

See table S1B for further detail on the qualitative assumptions behind this model.

2.2 Physical, natural and cultural capital

Now we couple the total cultural capital in the social-ecological system K_C, as defined in the main text, into the model of the previous section. We use the equations

$$\frac{dk_P}{dt} = s(k_P)E(K_N)f(k_P) - (\delta_P + n)k_P$$
$$\frac{dK_N}{dt} = T(K_C)G(K_N)L(k_P) - \delta_N K_N$$
$$\frac{dK_C}{dt} = P(K_N) - \delta_C K_C$$

where the new functions have the form

$$P(K_N) = \frac{P_0 K_N}{P_N + K_N}$$
$$T(K_C) = T_0 K_C$$

Cultural capital, in analogy to the other two capitals, is given a simple growth-decay form. Practice of traditional activities $P(K_N)$ associated with natural capital K_N serves to increase cultural capital, while cultural capital also degrades at rate δ_C if practices are not maintained. The growth function $P(K_N)$ is given a simple saturating form to ensure cultural capital in the model does not grow without limit. Like natural capital, we assume that it is the total cultural capital K_C shared by the people in the social-ecological system, rather than per-capita cultural capital, that affects the dynamics of the social-ecological system (in particular, the degree to which traditional practice supports natural capital).

In this model, the traditional practice associated with cultural capital is assumed to enhance the growth rate of natural capital according to the function $T(K_C)$. We use a linear function for $T(K_C)$ as the simplest possible coupling between K_C and K_N.

Parameters: As in previous section, with additionally $T_0 = 0.35$, $P_0 = 2$, $P_N = 2.5$, $\delta_C = 0.5$. These parameters were chosen:

- To ensure that there is a non-trivial poor attractor (that is, an attractor that is below the threshold in savings rate $k_P = d$ other than the attractor at zero physical, natural and cultural capital)

- To ensure that the same change to A as in the conventional poverty trap model (i.e. $A = 10$ to 16), representing a type II intervention, ensures that the poor attractor disappears.
- To explore new dynamics introduced by cultural capital. In the limit of slow dynamics of cultural capital, $\delta_C \to 0$ and $P_0 \to 0$, the role of cultural capital is effectively removed and model dynamics are the same as in the previous section.

See table S1C for further detail on the qualitative assumptions behind this model.

2.3 After type III alleviation strategy

We use the same model as in the section above but a new functional form for $L(k_P)$

$$L_2(k_P) = \rho + \frac{1-\rho}{1 + c_1 k_P^{c_2}}$$

At $\rho = 0$, $L_2(k_P) = L(k_P)$. $\rho > 0$ represents the local-scale effects of a transformation where farmers can devote some of their land to maintaining traditional crops with traditional practices even at high levels of physical capital. In Fig. 3B, $\rho = 0.5$.

See table S1D for further detail on the qualitative assumptions behind this model.

3 Subsistence trap model

For the subsistence trap model, we couple physical and natural capital according to the model

$$\frac{dk_P}{dt} = sf(k_P)E(K_N) - (\delta_P + n)k_P$$
$$\frac{dK_N}{dt} = G_2(K_N) - K_N D(k_P)$$

where existing functions are defined as above and the new functions are given by

$$G_2(K_N) = rK_N(K - K_N)$$
$$D(k_P) = \frac{D_0}{1 + e^{D_1(k_P - D_2)}}$$

In this model, physical capital per capita is modelled as before but without the feedbacks associated with variable savings rate. The growth rate of natural capital

is given by a simple logistic growth function $G_2(K_N)$. According to the assumptions used to create this model (see main text, table S1), natural capital is additionally degraded at low levels of per-capita physical capital. Natural capital is lost at a rate $D(k_P)$, which is given a sigmoidal form which has value D_0 for $k_p \lesssim D_2$ and zero for $k_p \gtrsim D_2$.

Parameters used were $s = 0.2$, $A = 10$, $q = 0.6$, $\alpha_1 = \alpha_2 = 0.4$, $\delta_P = 0.5$, $n = 0.5$, $r = 0.2$, $K = 5$, $D_0 = 0.8$, $D_1 = 4$, $D_2 = 2.5$. These parameters were chosen:

- To maintain similar parameters as in the intensification trap model (as far as possible)
- To ensure the existence of multiple (i.e. both poor and non-poor) attractors.

In the type II intervention, we increased r by 50% to $r = 0.3$, in order to model water innovations that improved the efficiency with which rainfall was used for irrigation, thereby increasing the rate at which natural capital was renewed.

See table S1E for further detail on the qualitative assumptions behind this model.

table S1. Qualitative model assumptions. Assumptions on which we base the poverty trap models. Note that the assumptions regard the properties of individual processes in the model. We do not anticipate here the emergent outcome of the model from the interaction of the processes. For example, the assumption 'natural capital can replenish itself' does not assume that natural capital will always return to its initial level. Instead, there is a process by which 'natural capital can replenish itself' and another by which 'natural capital can degrade'; the dynamics of natural capital is a result of the relative strengths of these two processes.

Assumption	Description or Examples	Model implementation
**A. Savings trap model**		
Production increases physical capital	Standard assumption in Solow model: part of production output is saved and invested (*71*).	$s(k_P)f(k_P)$
Physical capital increases production	Definition of physical capital	$f(k_P)$
Depreciation and population growth decrease physical capital per capita	Standard assumption of the Solow model (*71*)	n, δ_P
Savings rate steps up at a threshold of physical capital	Assumption of the savings trap model (*45*)	$s(k_P)$
**B. Intensification trap model with physical and natural capital:** As in **A**, and		
Natural capital can renew	Soil organic matter continually renewed by crop growth (*98*)<br>Biodiversity can recover from stresses<br>Seed can be rapidly multiplied	$G(K_N)$
Natural capital can degrade	Erosion, leaching, decomposition of soil organic matter (*98*)<br>Seeds lost if not planted	δ_N
Natural capital renewal rate decreases with increasing physical capital	'Intensification degrades' and 'Poor people do not degrade' assumptions from Table 1	$L(k_P)$
Natural capital increases production	Soil carbon increases production (*99*)<br>Agrobiodiversity increases agricultural production (*100*)<br>Pollination benefits from off-farm biodiversity increases production (*43*)	$E(K_N)$
**C. Intensification trap model with physical, natural and cultural capital:** As in **B**, and		
Cultural capital is increased through traditional practice	Engaging in these 'everyday' practices maintains knowledge (*42*, *44*)	$P(K_N)$
Cultural capital decreases over time if traditional practices are not undertaken	See above	δ_C
Natural capital increases cultural capital	Cultural and biological diversity co-evolve. Traditional practice emerges from interaction of cultural and natural capitals. If natural capital does not exist, there is no way to practice on it (*31*)	$P(K_N)$
Cultural capital helps produce natural capital	Natural capital in the form of agricultural biodiversity and corresponding ecosystem services is produced by humans through seed saving and sharing, grafting and landscape stewardship (*30*,*101*). See also 'Traditional knowledge and practice conserve the environment' assumption in Table 1.	$T(K_C)$
**D. Intensification trap model under *transform the system* (type III) pathway:** As in **C**, and		
Farmers can devote some of their land to maintaining traditional crops even under high levels of physical capital	Alleviation pathways based for example on food sovereignty (*17*)	$L_2(k_P)$

**E. Subsistence trap model**		
Production increases physical capital	Standard assumption in Solow model: part of production output is saved and invested (*71*).	$f(k_P)$
Physical capital increases production	Definition of physical capital	$f(k_P)$
Depreciation and population growth decrease physical capital per capita	Standard assumption of the Solow model (*71*)	n, δ_P
Natural capital can renew	Soil organic matter continually renewed by crop growth (*98*)<br>Biodiversity can recover from stresses<br>Seed can be rapidly multiplied	$G_2(K_N)$
Low physical capital is associated with increased degradation of natural capital	'Poor people degrade the environment' and 'intensification does not degrade' assumptions from Table 1	$D(k_P)$
Natural capital increases production	Soil carbon increases production (*99*)<br>Agrobiodiversity increases agricultural production (*100*)<br>Pollination benefits from off-farm biodiversity increases production (*43*)	$E(K_N)$
